# Esophageal Achalasia: An Unusual Cause of Respiratory Distress

**DOI:** 10.1155/crcc/1829559

**Published:** 2025-02-19

**Authors:** Océane Thiry, Marion Schaefer, Aurélie Cravoisy, Lionel Nace, Thibault François, Jacques Allard, Sébastien Gibot

**Affiliations:** ^1^Intensive Care Medicine, Hôpital Central, CHRU Nancy, Nancy, France; ^2^Gastroenterology and Hepatology, Hôpital Brabois, CHRU Nancy, Nancy, France

**Keywords:** endoscopic treatment, esophageal achalasia, respiratory distress

## Abstract

Esophageal achalasia is a rare condition affecting both men and women. It is a motility disorder of the esophagus resulting in the lack of relaxation of the lower sphincter, yielding to food stasis. While the condition typically presents with dysphagia and regurgitation, advanced stages may lead to severe complications including dyspnea and even acute respiratory distress. We herein report the case of a patient admitted to an intensive care unit following emergency prehospital management for acute respiratory failure and hemodynamic instability. The patient required invasive mechanical ventilation and vasopressor support due to tracheal compression and involvement of the cardiac, arterial, and venous structures. The diagnosis of esophageal achalasia was initially suspected. Based on CT, endoscopic esophageal decompression was performed, the situation dramatically improved, and diagnosis was subsequently confirmed by esophageal manometry.

## 1. Introduction

Esophageal achalasia is a primary motility disorder of the esophagus of unknown etiology characterized by the absence of relaxation of the lower esophageal sphincter (LES) and the absence of normal peristaltic contractions in the esophageal body. These alterations in peristalsis results from the loss of ganglion cells within the myenteric plexus of Auerbach, located in the smooth muscle layer of the esophagus and LES. Overtime, the esophagus becomes paralyzed and progressively dilates, losing its ability to propel food into the stomach, leading to food stasis. Esophageal achalasia is a rare disease with an annual incidence of approximately 2 per 100,000 individuals and a prevalence of 27 per 100,000. Esophageal achalasia affects both sexes equally and can occur at any age [1]. The hallmark symptom is dysphagia described as a sensation of food obstruction or discomfort during its progression, at the retrosternal level. In severe cases, the disorder may lead to dyspnea, and exceptionally, to acute respiratory distress [2]. We herein describe a severe esophageal achalasia revealed by a life-threatening acute respiratory failure.

## 2. Case Report

### 2.1. Presentation

A 51-year-old female patient was admitted to this intensive care unit (ICU) for acute respiratory failure. The patient had been in her usual state of health until she suddenly presented an episode of dyspnea and agitation at home, prompting the activation of emergency medical support service. Her medical history was only remarkable for multiple, brief, and spontaneous resolutive episodes of acute dyspnea over the past 5 years. Medical advice was sought several times and reportedly concluded to panic attacks.

On examination, the patient was agitated, cyanotic, and dyspneic with diffuse wheezing and rhonchi on auscultation. The temporal temperature was 36.7°C, blood pressure 160/105 mmHg, pulse 110 bpm, respiratory rate 34 breaths per minute, and oxygen saturation 94% while the patient was receiving 15 L/min of supplemental oxygen. Blood glucose level was within the normal range. She rapidly deteriorated to comatose state (Glasgow Coma Score of 9). Endotracheal intubation was performed under induction with etomidate and suxamethonium followed by continuous sedation with midazolam and sufentanil. End-tidal carbon dioxide was measured at 90 mmHg. A diagnosis of bronchospasm was suspected, and the patient was administered 120 mg of methylprednisolone before transfer to this ICU.

Upon ICU admission, mechanical ventilation was noticed to be difficult because of very high peak inspiratory pressures (> 60 cm H_2_O) despite adequate sedation, muscle paralysis, and careful respirator setting. Moreover, the patient developed hemodynamic instability, requiring volume resuscitation and norepinephrine infusion.

Arterial blood gas analysis revealed respiratory acidosis: pH 7.14, PaCO_2_ 78 mmHg, PaO_2_ 286 mmHg, lactate 0.8 mmol/L at FiO_2_ = 1. Hypokalemia at 2.3 mmol/L and hypoprotidemia at 44 g/L were the only noticeable biological abnormalities.

**Figure 1:**
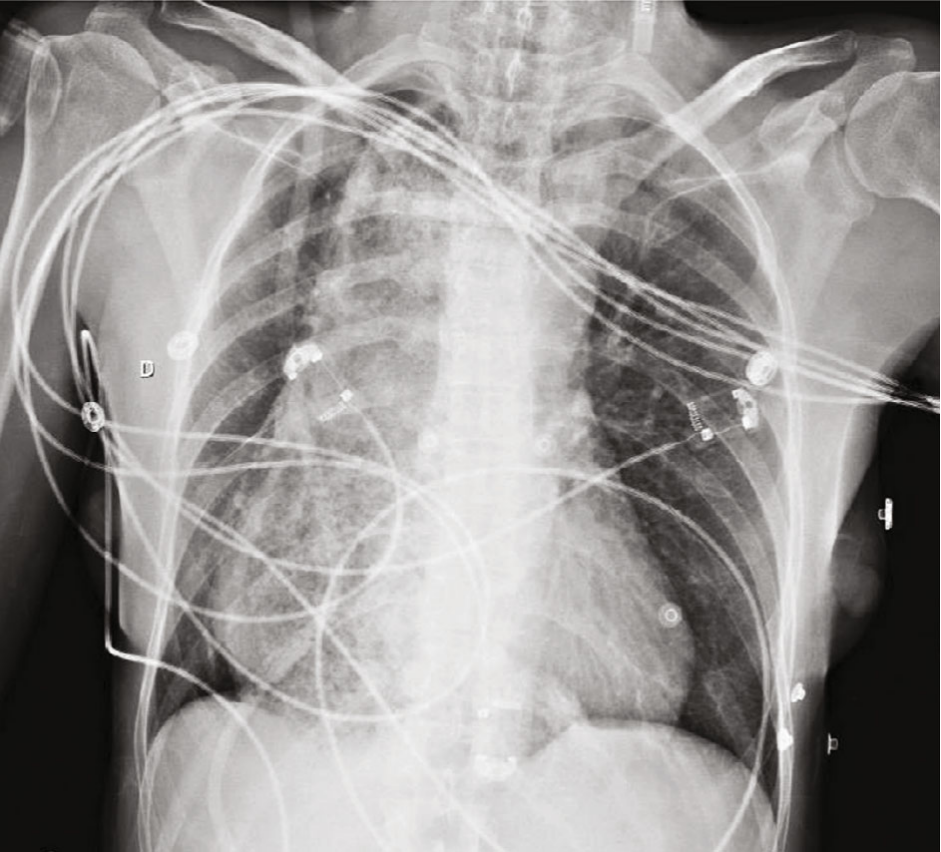
Chest X-ray on admission.

A chest X-ray was immediately performed and revealed a large right thoracic mass that was difficult to characterize (Figure 1). Attempts at bronchoscopy were unsuccessful due to external compression preventing bronchoscope from passing beyond the carina.

**Figure 2:**
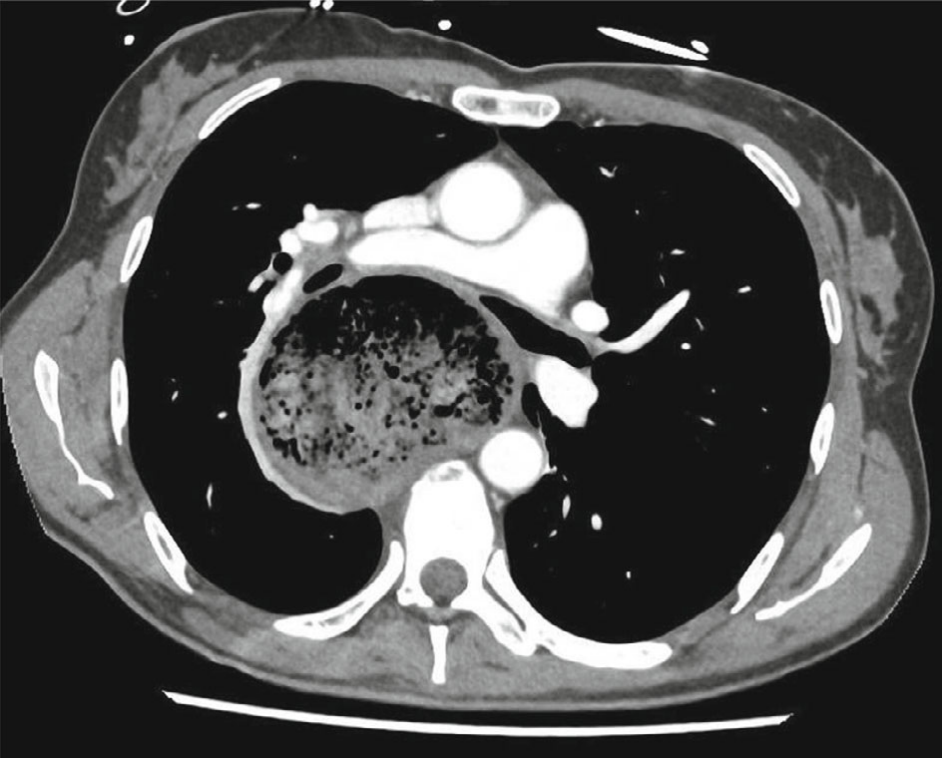
CT scan on admission.

A CT scan was then performed and revealed esophageal distension, measuring up to 9 cm in diameter, filled with food stasis. The dissension was caused by a marked stenosis of the esogastric junction, resulting in a major mass effect on the trachea just above the carina, with near-complete airway collapse. Additionally, there was a compression of the arterial brachiocephalic trunk, superior vena cava, the right and left pulmonary arteries, pulmonary veins, and left atrium (Figure 2).

### 2.2. Management

Fluids, norepinephrine, sedation, and curare were continued. Amoxicillin clavulanate was administered because of a high suspicion of inhalation pneumonia, and potassium and polyvitamins were administered to address hypokalemia and potential nutritional deficiencies. Upper gastrointestinal endoscopy revealed a severely distended esophagus, almost completely filled with solid food. Endoscopic debridement was thus performed to remove the obstructing food material. During this procedure, mechanical ventilation parameters improved significantly, with peak inspiratory pressures decreasing to 30 cm H_2_O, and plateau pressure to 12 cm H_2_O. Concurrently, hemodynamics stability was restored, allowing for the discontinuation of norepinephrine.

### 2.3. Follow-up

The patient was successfully extubated the following day without complications. During subsequent discussion, she reported experiencing “feeding difficulties” during the previous week, including a sensation of incomplete emptying and frequent regurgitations. An exclusive liquid diet was initiated, and the patient was discharged from the ICU after 2 days.

Further evaluation with esophageal manometry was attempted; however, the probe could not be passed across the esophagogastric junction (EGJ) due to the presence of a significantly dilated megaesophagus. The diagnosis of Type 1 achalasia was established based on the combination of complete aperistalsis observed during manometry, findings from the CT scan and endoscopy without organic stricture, and the patient's clinical history. Two weeks later, the patient underwent a peroral endoscopic myotomy (POEM), which was successfully performed. She was discharged and resumed her daily activities shortly thereafter and transitioned to a normal diet 10 days after the procedure.

## 3. Discussion

Esophageal achalasia is a rare primary motility disorder characterized by insufficient relaxation of the LES in combination with absent peristalsis. Type I achalasia is diagnosed in case of abnormal median integrated relaxation pressure (IRP) of the EGJ and 100% failed peristalsis. Type II and III achalasia are related to abnormal median IRP and 100% failed peristalsis associated with 20% or more swallows with panesophageal pressurization, or abnormal median IRP and 20% or more swallows with premature/spastic contraction and no evidence of peristalsis, respectively. Dysphagia is the cardinal symptom, present in over 90% of cases, and typically affects both solids and liquids.

Respiratory symptoms are relatively common in patients with achalasia, reported in approximately 40% of cases, and may include cough, hoarseness, wheezing, and, less frequently, shortness of breath [3]. These symptoms are probably secondary to regurgitation and inhalation of the esophageal contents. In our patient, repeated episodes of acute dyspnea over the past 5 years were brief (less than 30 minutes), spontaneously resolutive and misattributed to panic attacks, as dysphagia – often the most prominent symptom – was notably absent. This atypical presentation highlights the diagnostic challenges in such cases.

In advanced stages, severe esophageal dilation (megaesophagus) can result in extrinsic compression of adjacent structures, including the trachea and left atrium. Tracheal compression, severe enough to cause acute respiratory distress requiring intubation and mechanical ventilation, as described in this case report, is very uncommon [4, 5]. Exceptionally, esophageal dilation may become large enough as to compress the left atrium and cause hemodynamic instability [6].

Endoscopic decompression of the esophagus is the treatment of choice, relieving extrinsic compression on the trachea and heart, thus restoring respiratory and circulatory functions.

The formal diagnosis of achalasia is typically made through esophageal manometry, which demonstrated aperistalsis and impaired LES relaxation. However, in this case, severe esophageal dilatation precluded successful manometry due to the inability to advance the probe across the EGJ. Instead, the diagnosis was based on a combination of complete aperistalsis and imaging, endoscopic, and clinical presentation.

Although no therapy can restore peristalsis, treatment of achalasia aims to reduce LES pressure. Treatment includes endoscopic or surgical interventions. In the presented case, the patient underwent POEM, a minimally invasive procedure first described in 2010. This endoscopic intervention consists of endoscopic section of the muscular layer of the lower esophagus and LES through a mucosal incision and tunneling in the submucosa [7]. This approach offers similar results than laparoscopic Heller's myotomy that used to be the standard of care [8]. Given the very low complication rate and its superior efficacy compared to pneumatic dilation of the LES, POEM is gradually replacing pneumatic dilation. The minimally invasive approach, with liquid refeeding on Day 1 and a return to a normal diet between Days 7 and 10, combined with the short procedure duration and efficacy comparable to Heller's myotomy, leads our center to offer POEM as the first-line treatment for patients with achalasia.

## 4. Conclusion

Acute respiratory failure revealing esophageal achalasia is a rare and life-threatening complication due to external compression of the trachea by a dilated esophagus. Early recognition is crucial to expedite the salvaging endoscopic treatment.

## Data Availability

All pertinent data are included in the article. Further inquiries can be directed to the corresponding author.
